# Quantifying brain metabolism from FDG‐PET images into a probability of Alzheimer's dementia score

**DOI:** 10.1002/hbm.24783

**Published:** 2019-09-10

**Authors:** Evangeline Yee, Karteek Popuri, Mirza Faisal Beg, the Alzheimer's Disease Neuroimaging Initiative

**Affiliations:** ^1^ School of Engineering Science Simon Fraser University Burnaby BC Canada

**Keywords:** 3D CNN, dementia of Alzheimer's type (DAT), FDG‐PET

## Abstract

^18^F‐fluorodeoxyglucose positron emission tomography (FDG‐PET) enables in‐vivo capture of the topographic metabolism patterns in the brain. These images have shown great promise in revealing the altered metabolism patterns in Alzheimer's disease (AD). The AD pathology is progressive, and leads to structural and functional alterations that lie on a continuum. There is a need to quantify the altered metabolism patterns that exist on a continuum into a simple measure. This work proposes a 3D convolutional neural network with residual connections that generates a probability score useful for interpreting the FDG‐PET images along the continuum of AD. This network is trained and tested on images of stable normal control and stable Dementia of the Alzheimer's type (sDAT) subjects, achieving an AUC of 0.976 via repeated fivefold cross‐validation. An independent test set consisting of images in between the two extreme ends of the DAT spectrum is used to further test the generalization performance of the network. Classification performance of 0.811 AUC is achieved in the task of predicting conversion of mild cognitive impairment to DAT for conversion time of 0–3 years. The saliency and class activation maps, which highlight the regions of the brain that are most important to the classification task, implicate many known regions affected by DAT including the posterior cingulate cortex, precuneus, and hippocampus.

## 1 INTRODUCTION

Dementia of the Alzheimer's type (DAT) used to be regarded as a disease with discrete clinical stages. In 2011, the National Institute on Aging–Alzheimer Association (NIA–AA) created diagnostic recommendations for three distinct stages: preclinical DAT, mild cognitive impairment (MCI), and DAT (McKhann et al., 2011). Preclinical DAT was defined for individuals without overt cognitive symptoms, and MCI was defined for individuals with noticeable cognitive decline. In recent years, longitudinal studies have shown that the cognitive decline in DAT is a continuous process that takes place over a long period of time, and that the pathological changes of DAT are also part of a continuous process that begins decades before the appearance of cognitive symptoms (Fagan et al., 2014; Monsell et al., 2014; Resnick et al., 2010; Sutphen et al., 2015; Villemagne et al., 2011). These discoveries have prompted a shift to conceptualize DAT as a continuum rather than three distinct stages (Dubois et al., 2016; Jack et al., 2018). Applying the continuum concept of DAT to automated image‐based interpretation is challenging. It requires methods to not only discriminate between images from the extreme ends of the DAT spectrum, but also generalize to images along the entire spectrum.

^18^F‐fluorodeoxyglucose positron emission tomography (FDG‐PET) plays a major role in the diagnosis of DAT through its capacity to detect early abnormalities in brain metabolism (Reiman et al., 2004). Most of the earlier FDGPET classification studies were trained and evaluated on images from the extreme ends of the DAT spectrum: normal control (NC) and DAT. One recent work has demonstrated the ability of FDG‐PET classification to generalize its predictive performance to images along the entire DAT spectrum (Popuri et al., 2018). The most common approach used to extract DAT‐related patterns from FDG‐PET images is region‐of‐interest (ROI) approach (Gray et al., 2011; Lu et al., 2018; Pagani et al., 2015; Pagani et al., 2017; Popuri et al., 2018; Toussaint et al., 2012). In a ROI‐based approach, a subject's FDG‐PET image is registered to a corresponding structural MRI image or a custom FDG‐PET template, then the mean intensity in each predefined ROI is extracted and fed into classifiers such as support vector machines. However, the complex spatiotemporal pattern of DAT‐related abnormalities is not likely to be fully captured by measuring the intensities of a limited number of ROIs defined based on a priori assumptions (Fan, Resnick, Wu, & Davatzikos, 2008). ROI‐based approaches also require accurate segmentation and registration, both of which are computationally intensive and time consuming. These steps can potentially introduce errors in assessing ROI‐based metabolism measures, especially in the presence of structural atrophy. Other methods include voxel‐based approach where the registered FDG‐PET image is analyzed on a voxel‐by‐voxel basis using statistical methods such as *t* test (Arbizu et al., 2013). ROI‐based and voxel‐based approaches often require the use of individual MRI images to superimpose structural ROIs on FDG‐PET images or the use of a custom FDG‐PET template to register FDG‐PET images to a common space. However, a structural MRI image may not be available for every subject and custom FDG‐PET template may be limited for use in specific populations. These limitations provide hurdles for computational algorithms to be useful in the clinical setting.

In this work, we developed a 3D convolutional neural network (CNN) and showed its predictive performance on images along the entire DAT spectrum. 3D CNN allows us to make predictions using only FDG‐PET images without defining any a‐priori ROIs. Recently, 3D CNNs have been shown to be effective in various medical imaging applications. These applications include the detection of microbleeds from MRI, detection of pulmonary nodules from computed tomography (CT), segmentation of the liver from CT, segmentation of vertebral bodies from MRI, segmentation of brain lesions from MRI, and segmentation of subcortical structures from MRI (Dolz, Desrosiers, & Ayed, 2017; Dou, Chen, Jin, et al., 2016; Dou, Chen, Yu, et al., 2016; Kamnitsas et al., 2017; Korez, Likar, Pernus, & Vrtovec, 2016; Zhu, Liu, Fan, & Xie, 2018). Most relevant to our work, 3D CNNs have shown success in the classification of DAT using MRI (Hosseini‐Asl, Gimel'farb, & El‐Baz, 2016; Payan & Montana, 2015). For FDG‐PET, however, existing deep learning studies have employed 2D CNNs which do not take full advantage of the spatial topographic patterns inherent in FDG‐PET images (Ding et al., 2018; Liu, Cheng, & Yan, 2018). Neural networks are often described as black boxes. This has given rise to concern surrounding the transparency and interpretability of neural networks. Besides presenting a 3D CNN with high predictive performance and strong generalizability, we explained how our 3D CNN model makes a prediction by visualizing the saliency and class activation maps.

## 2 MATERIALS

### 2.1 Data

Data used in the preparation of this article were obtained from the Alzheimer's Disease Neuroimaging Initiative (ADNI) database (http://adni.loni.usc.edu). ADNI was launched in 2003 as a public–private partnership, led by principal investigator Michael W. Weiner, MD. The primary goal of ADNI has been to test whether serial MRI, PET, other biological markers, and clinical and neuropsychological assessment can be combined to measure the progression of MCI and early Alzheimer's disease (AD). Full details of subject recruitment, scanning protocols, and diagnostic criteria are available on http://www.adni-info.org.

### 2.2 Database stratification

Using the stratification scheme proposed by Popuri et al. (2018), we stratified the NC, MCI, and DAT groups into seven subgroups: sNC (stable NC, remained NC throughout), uNC (unstable NC, converted to MCI in the future), pNC (progressive NC, progressed to DAT in the future), sMCI (stable MCI), pMCI (progressed to DAT in the future), eDAT (converted to DAT during ADNI window), and sDAT (joined ADNI with clinical diagnosis of DAT). These subgroups represent the DAT− and DAT+ trajectories of future disease progression. Subjects with clinical diagnosis of DAT at follow‐ups regardless of their diagnosis at baseline are considered to be on the DAT+ trajectory. Thus, the pNC, pMCI, eDAT, and sDAT subgroups are considered to be on the DAT+ trajectory. These images are associated with a future diagnosis of DAT. The sNC, uNC, and sMCI subgroups do not have a future designation of DAT (in the follow‐ups available) and hence these subgroups are deemed to be on the DAT− trajectory. Demographic details of all subgroups are presented in Table 1. We used the baseline and longitudinal images of 359 sNC and 237 sDAT subjects for network training. In total, we used 752 sNC images and 459 sDAT images for network training and evaluation. We used all images of the subjects in the uNC, sMCI, pNC, and pMCI subgroups as an independent test set to assess the generalizability of our network.

**Table 1:** Demographic details of ADNI subjects

Dementia trajectory	Group	Number of participants	Number of images	Gender (F/M)	Age	MMSE
DAT−	sNC	359	752	192/167	75.44 ± 5.95	29.08 ± 1.17
DAT−	uNC	51	108	20/31	78.98 ± 4.90	29.06 ± 1.14
DAT−	sMCI	427	871	173/254	74.94 ± 7.74	27.85 ± 1.96
DAT+	pNC	19	56	9/10	78.05 ± 4.52	28.91 ± 1.21
DAT+	pMCI	210	496	91/119	75.02 ± 7.16	26.80 ± 2.05
DAT+	eDAT	135	239	54/81	76.64 ± 6.67	22.27 ± 4.45
DAT+	sDAT	237	459	97/140	75.75 ± 7.51	22.01 ± 3.64

Abbreviations: ADNI, Alzheimer's Disease Neuroimaging Initiative; MCI, mild cognitive impairment; pMCI, progressive MCI; pNC, progressive NC; sDAT, stable Dementia of the Alzheimer's type; sMCI, stable MCI; sNC, stable normal control; uNC, unstable normal control.

### 2.3 FDG‐PET image preprocessing

We obtained preprocessed FDG‐PET images from the LONI Image Data Archive. Briefly, the ADNI FDG‐PET preprocessing steps include co‐registering the raw FDG‐PET frames, averaging the co‐registered frames, mapping the averaged image into a standard 160 × 160 × 96 image grid with 1.5 × 1.5 × 1.5 mm^3^ voxel size, performing intensity normalization such that the average intensity of foreground voxels is exactly one, and filtering the normalized image with a scanner‐specific filter function to produce an image with isotropic resolution of 8 mm FWHM. Full details of ADNI FDG‐PET preprocessing steps are available at (http://adni.loni.usc.edu/methods/pet-analysis). We registered the preprocessed images directly to the MNI template with 1.5 mm^3^ voxel size via 7‐parameter rigid transformation using FSL‐FLIRT software (Jenkinson, Bannister, Brady, & Smith, 2002; https://fsl.fmrib.ox.ac.uk/fsl/fslwiki/FLIRT). Note that this registration step does not account for atrophic differences; it was used simply to correct for pose differences, as our convolutional neural network is not rotation invariant. We performed min–max scaling to rescale the image intensity values to the range between 0 and 1.

## 3 METHODS

Our proposed network is a 3D CNN with residual connections that takes a 3D FDG‐PET image as input and outputs a DAT probability score with 1 representing the highest probability of the image belonging to the DAT class, and 0 representing the control (normal aging) class.

### 3.1 Network architecture

**Figure 1:**
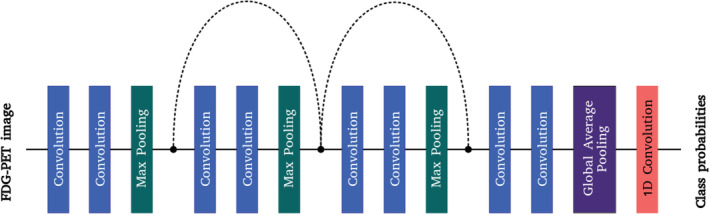
3D fully‐convolutional network with shortcut connections. The dotted shortcut connections match the output dimensions of the max pooling layers by performing 1 × 1 × 1 convolution with a stride of 2

Our 3D CNN has a total of eight convolutional layers. The number of filters used in each convolutional layer is 2, 4, 16, 16, 64, 64, 72, and 96, respectively. Figure 1 illustrates our network architecture. The first layer is a convolutional layer with a kernel size of 5 × 5 × 5 and a stride of 2 which reduces the input spatial dimensions and subsequently lowers the memory usage. The second layer is a convolutional layer with a kernel size of 3 × 3 × 3 and a stride of 1, followed by a max pooling layer with a kernel size of 3 × 3 × 3 and a stride of 2. Next, we used two residual learning blocks to learn hierarchical features. Residual learning block was first introduced by He, Zhang, Ren, and Sun (2016a) to address the degradation problem in deep learning where adding more layers leads to higher training error and rapid performance degradation. Each residual learning block consists of two convolutional layers and a shortcut connection that bypasses the convolutional layers. The shortcut connection creates identity mapping such that the output of a residual learning block is the element‐wise addition of its input and the output of its last convolutional layer. In a series of residual learning blocks, the shortcut connections allow information to propagate more easily. An important and practical feature of residual learning blocks is that they are computationally efficient. The shortcut connection can be used without introducing additional parameters. For each residual learning block, we added a 3 × 3 × 3 max pooling layer with a stride of 2 after its last convolutional layer, and we also added a 1 × 1 × 1 convolutional layer with a stride of 2 to the shortcut connection. Following the residual learning blocks, we used two convolutional layers with a kernel size of 3 × 3 × 3 and a stride of 1 to learn high‐level features.

We added an instance normalization (IN) layer and leaky rectified linear units (ReLU) after each of the aforementioned convolutional layers not within a residual block (Maas, Hannun, & Ng, 2013; Ulyanov, Vedaldi, & Lempitsky, 2017). Normalization layers help improve training convergence speed. Instance normalization layers normalize each feature map of each channel of each training sample using the sample mean and variance. The widely adopted batch normalization (BN) layer, on the other hand, normalizes each feature map of each channel of a mini‐batch of data using the mini‐batch mean and variance. Thus, batch normalization requires a larger batch size in order to accurately estimate the mini‐batch mean and variance. Training with a larger batch size is memory intensive and may lead to lower generalizability (Keskar, Mudigere, Nocedal, Smelyanskiy, & Tang, 2016). Within the residual blocks, we added instance normalization and leaky ReLU before each convolutional layer as preactivation (He, Zhang, Ren, & Sun, 2016b).

The final classification layers consist of a global average pooling layer and a 1 × 1 convolutional layer with softmax activation. Conventional CNN uses a flattening layer to vectorize the feature maps of the last convolutional layer and adds fully connected layers on top of the long 1D vector. The combination of a flattening layer and fully connected layers results in a large number of parameters. This kind of network is prone to overfitting. We replaced the flattening layer with a global average pooling layer which has been shown to act as a regularizer (Lin, Chen, & Yan, 2013). The global average pooling layer simply computes the average intensity of each feature map. The final 1 × 1 convolutional layer acts as a fully connected layer. Table 2 shows the details of our network architecture.

**Table 2:** Network architecture and parameters

Layers	Number of filters	Kernel size/kernel stride	Output dimension
Dropout (0.2)			121 × 145 × 121
Convolution	2	5 × 5 × 5/2 × 2 × 2	61 × 73 × 61
Dropout (0.2)			61 × 73 × 61
Convolution	4	3 × 3 × 3/1 × 1 × 1	61 × 73 × 61
Max pooling		3 × 3 × 3/2 × 2 × 2	31 × 37 × 31
Residual block 1/convolution	16	3 × 3 × 3/1 × 1 × 1	31 × 37 × 31
Residual block 1/convolution	16	3 × 3 × 3/1 × 1 × 1	31 × 37 × 31
Residual block 1/max pooling		3 × 3 × 3/2 × 2 × 2	16 × 19 × 16
Residual block 2/convolution	64	3 × 3 × 3/1 × 1 × 1	16 × 19 × 16
Residual block 2/convolution	64	3 × 3 × 3/1 × 1 × 1	16 × 19 × 16
Residual block 2/max pooling		3 × 3 × 3/2 × 2 × 2	8 × 10 × 8
Dropout (0.2)			8 × 10 × 8
Convolution	72	3 × 3 × 3/1 × 1 × 1	8 × 10 × 8
Dropout (0.2)			8 × 10 × 8
Convolution	96	3 × 3 × 3/1 × 1 × 1	8 × 10 × 8
Global average pooling			96
Convolution	2	1 × 1/1 × 1	2
Total number of trainable parameters	460,216		

### 3.2 Network training

To handle class imbalance, we optimized the weighted binary cross‐entropy loss given by:

$$L\text{cross}-\text{entropy}=C0\left(1-y\right)\log\left(p\left(\tilde{y}=0|X\right)\right)+C1y\log\left(p\left(\tilde{y}=1|X\right)\right)$$

where *C*_0_ and *C*_1_ are, respectively, the sNC and sDAT class weights, and *p*($\tilde{y}$ = 0|*X*) and *p*($\tilde{y}$ = 1|*X*) are, respectively, the NC and DAT class probabilities given an input image *X*. The class weights are computed using:

$$C0=\frac{N_{\text{sDAT}}}{N_{\mathrm{sNC}}+N_{\text{sDAT}}}$$

$$C1=\frac{N_{\mathrm{sNC}}}{N_{\mathrm{sNC}}+N_{\text{sDAT}}}$$

where *N*_*sNC*_ and *N*_*sDAT*_ are the number of sNC and sDAT training images. We incorporated *L* ^2^ regularization in the classification layer to help reduce overfitting. The *L* ^2^ loss is formulated as:

$$L_{\text{regularization}}=\lambda {\left\|w\right\|}^{2}$$

where *λ* = 0.01 is a hyperparameter representing the influence of regularization, and *w* is the weight vector of the final convolutional layer. The total loss is then given by:

$$L=L_{\text{cross}-\text{entropy}}+L_{\text{regularization}}$$

We used dropout at several layers in the network to further reduce overfitting (Srivastava, Hinton, Krizhevsky, Sutskever, & Salakhutdinov, 2014). The dropout layer randomly sets a fraction of the input voxels to zero during training, forcing the network to learn more robust features. We augmented our training data by applying left–right flip, rotation, and translation. Initially, we performed data augmentation on‐the‐fly which allowed us to train the network using a large number of unique images. However, 3D rotation of the input volume increases both the training time and graphical processing unit (GPU) memory usage. We opted to generate a fixed number of rotated images by applying 5° rotation around each of the three axes. We also generated spatially normalized images by co‐registering the ADNI preprocessed FDG‐PET to the corresponding MRI, registering the MRI to the MNI space via 12‐parameter affine registration, and applying the MRI‐to‐MNI space transformation to the co‐registered FDG‐PET. These spatially normalized images were used to augment the training data and visualize the saliency and class activation maps. The left–right flip and translation of a maximum of five voxels in each axis were done on‐the‐fly. The 3D convolutional filters were initialized using the He‐weight initialization method (He, Zhang, Ren, & Sun, 2015). Our network was trained end‐to‐end using mini‐batches of size 8 and Adam optimizer with 0.001 learning rate and 0.9 momentum for a maximum of 50 epochs (Kingma & Ba, 2014). We monitored the validation loss and performance after every 2 epochs, stopping the training process early whenever the lowest validation loss stayed constant for 8 epochs.

### 3.3 Network visualization

In an effort to make our model transparent and interpretable, we utilized gradient‐based visualization techniques. Guided backpropagation computes the gradient of an output class probability with respect to the input image, which reflects how small changes in each input image pixel affect the output class probability (Springenberg, Dosovitskiy, Brox, & Riedmiller, 2014). Given an input image, we performed a forward pass to the DAT class probability node, and then back‐propagated the gradients to get a reconstructed image. During backpropagation, we set negative gradients to zero because negative gradients correspond to the deactivation of a higher convolutional layer, with the classification layer being the topmost layer. This helps to reconstruct an image that activates not just the neurons in the lower layers where general features are learned, but also the neurons in the higher convolutional layers where complex features are learned. This guided backpropagation approach has been shown to produce sharper and more accurate images (Springenberg et al., 2014). We smoothed the reconstructed image by applying a gaussian filter with a sigma of 2, and rescaled the intensity to the range between −1 and 1. A grand average saliency map was computed by taking the average of the saliency maps produced by performing guided backpropagation for every sNC and sDAT image.

One of the drawbacks of guided backpropagation is that the discontinuities in gradients through nonlinear leaky ReLU and max pooling layer may cause undesirable artifacts. Gradient‐weighted class activation mapping (GradCAM) uses the activation maps of a convolutional layer, usually the last convolutional layer, to localize regions in the input image that are of importance for predicting a target class (Selvaraju et al., 2017). Grad‐CAM avoids the gradient backpropagation problems by propagating the gradients of the output class probability node only until the last convolutional layer. These gradients capture the importance of each activation map for a target class and can be used to weigh the activation maps to generate a class‐discriminative heatmap. Given an input image, we forward propagated the image, and computed the gradients of the DAT probability node with respect to the feature maps of the convolutional layer before the global average pooling layer. We then performed global average pooling of the gradients to obtain a weight vector which represents the importance of each activation map. The class activation map was generated by computing the weighted linear combination of the activation maps. We then visualized the positive pixels in the class activation map by setting the negative pixels to zero, as previous experiments have shown that negative pixels in the class activation maps are more likely to be associated with nondesired classes (Selvaraju et al., 2017). A drawback of Grad‐CAM is that it produces down‐sampled and coarse class activation maps. The class activation map of size 8 × 10 × 8 was up‐sampled to the input image resolution using spline interpolation. The intensity of the up‐sampled class activation map was rescaled to the range between 0 and 1. We also computed Grad‐CAM for the NC class. Again, this process was repeated for every sNC and sDAT image to generate grand average class activation maps.

For network visualization, we used the spatially normalized FDG‐PET images as inputs to ensure that there is spatial correspondence between individual saliency and class activation maps. We co‐registered the FDG‐PET images to their corresponding MRI images. We then transformed the MRI images to MNI space via affine registration and applied the same transformation to the co‐registered FDG‐PET images to obtain spatially normalized FDG‐PET images.

### 3.4 Experiment

We trained on the baseline and longitudinal images to make full use of all the available sNC and sDAT data. Importantly, we created the training, validation, and test sets by splitting at the subject level. All images of a training subject were used for training, and similarly all images of a test subject were used for testing. Therefore, the training, validation, and test sets contained mutually exclusive subjects. Splitting at the image level could otherwise lead to biased and optimistic results, especially if some images from the same subject were used for training and other images from the same subject were used for testing.

We performed twice‐repeated stratified fivefold cross‐validation (CV) to evaluate our network. In fivefold cross‐validation, the data were split into fivefolds. Each time, one fold (20% of data) was set aside for testing, while one out of the remaining four folds was randomly selected as the validation set and the rest were used as training sets. We chose to perform fivefold cross‐validation instead of the typical 10‐fold cross‐validation because this allowed us to test on a larger number of images. We repeated the fivefold cross‐validation twice to obtain a better estimate of the classification performance. Altogether, we performed 10 iterations of training, validating, and testing on 60%, 20%, and 20% of sNC and sDAT subjects respectively, resulting in 10 networks. Since each network was only trained on a subset of the sNC and sDAT images, we created an ensemble by averaging the predictions from the 10 networks to make full use of the sNC and sDAT images. We tested the generalizability of our ensemble by testing on the unseen uNC, pNC, pMCI, and eDAT images.

## 4 RESULTS

### 4.1 Cross‐validation performance

**Figure 2:**
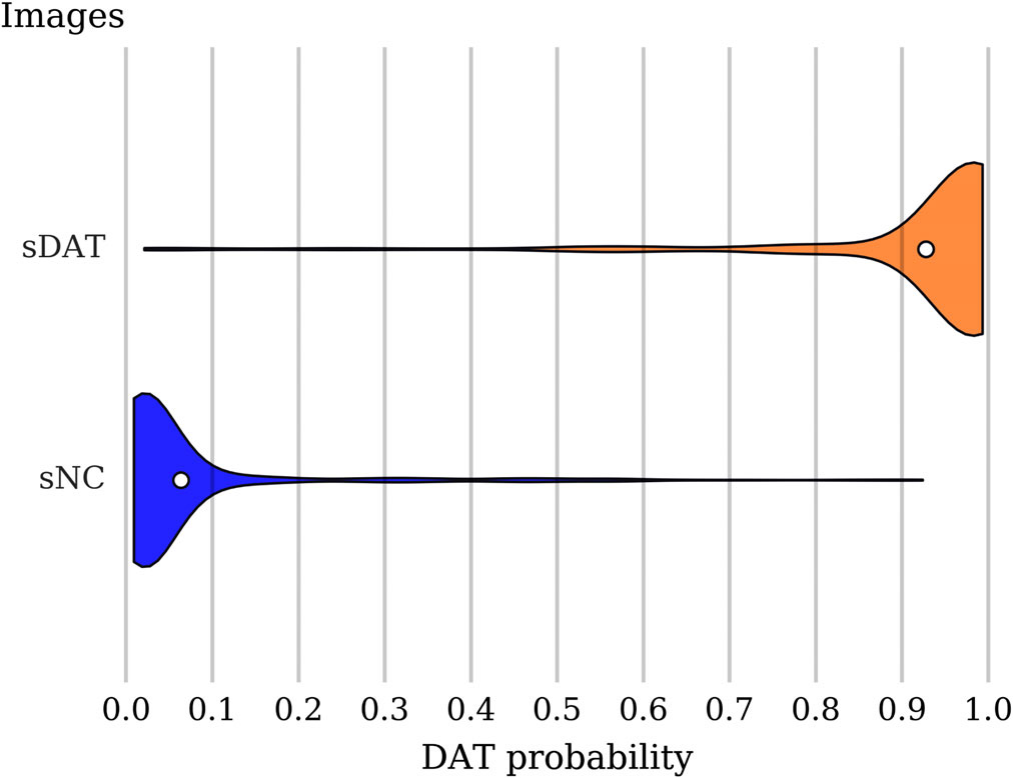
DAT probability score distribution among the sNC and sDAT test images. The violin plot shows the density (relative proportion of images), while the white dot gives the mean probability. A threshold of 0.5 leads to classification accuracy of 93.5%, sensitivity of 92.3%, specificity of 94.2%, and AUC of 0.976. sDAT, stable Dementia of the Alzheimer's type; sNC, stable normal control

In Table 3, we present the classification performance on the sNC and sDAT images averaged across the 10 test folds. The accuracy, sensitivity, and specificity were computed using a threshold of 0.5, with probability greater than 0.5 assigned to the DAT class. The results obtained show that our proposed method surpassed other competing published methods in AUC and accuracy. Figure 2 shows that the predicted DAT probability scores for the sNC and sDAT test images are clustered around mean values of 0.064 and 0.928, respectively.

**Table 3:** Comparison of published sNC versus sDAT classification performance

Study	sNC subjects	sDAT subjects	AUC	Accuracy (%)	Sensitivity (%)	Specificity (%)	Evaluation scheme
Herholz et al. (2002)	28	28	0.970	93.0	**93.0**	93.0	Independent test
Ishii et al. (2006)	15	15	0.967	93.0	93.0	93.0	Independent test
Haense, Herholz, Jagust, and Heiss (2009)	102	89	0.896	–	83.0	78.0	Independent test
Illan et al. (2011)	–	–	–	88.2	87.7	88.6	Independent test
Arbizu et al. (2013)	20	21	0.948	–	–	–	Independent test
Gray et al. (2011)	69	71	0.900	81.6	82.7	80.4	Repeated hold‐out
Toussaint et al. (2012)	80	80	–	91	84	**100**	Leave‐one‐out CV
Popuri et al. (2018)	360	238	0.954	89.8	87.0	91.7	Subagging
Zhang et al. (2011)	52	51	0.938	86.5	86.3	86.6	10‐fold CV
Liu et al. (2018)	100	93	0.953	91.2	91.4	91.0	10‐fold CV
Our method	359	237	**0.976**	**93.5**	92.3	94.2	Repeated fivefold CV

Abbreviations: sDAT, stable Dementia of the Alzheimer's type; sNC, stable normal control.

### 4.2 Generalizability to other images along the DAT spectrum

**Figure 3:**
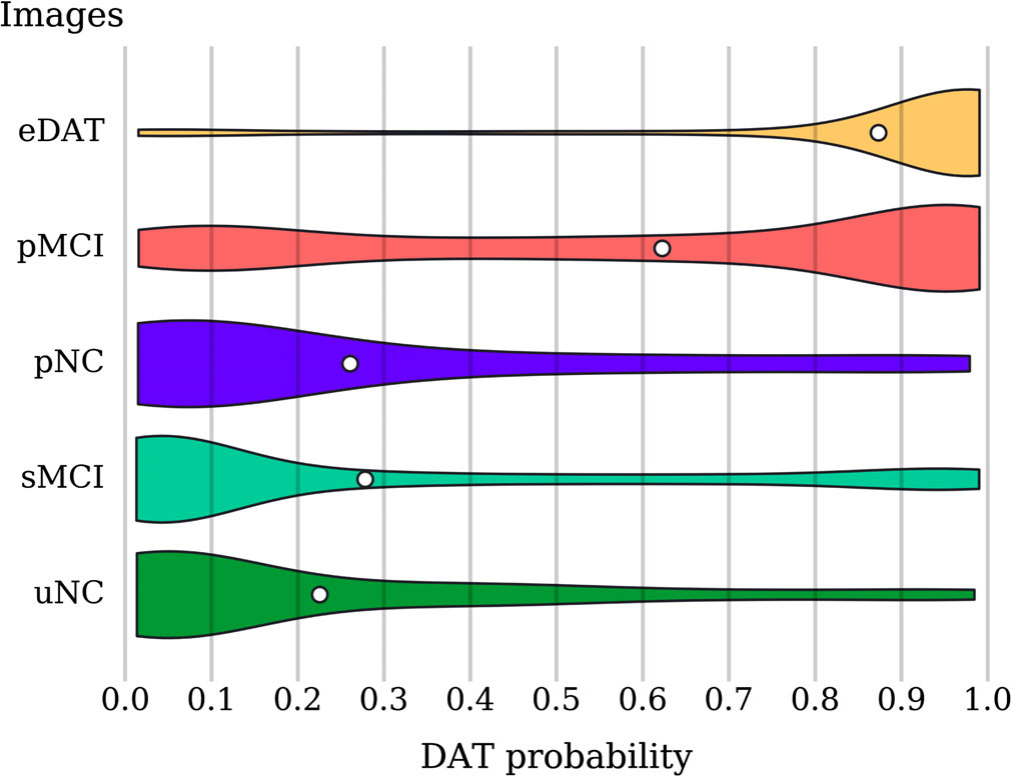
DAT probability score distribution among all the independent test images from the uNC, sMCI, pNC, pMCI, and eDAT subgroups. The violin plot shows the density (relative proportion of images), while the white dot gives the mean probability score. Most of the eDAT images are clustered around the high probability scores. The pMCI images exhibit bimodal clustering with a dominant cluster around higher probability scores and another cluster around lower probability scores. The pNC, sMCI, and uNC images are clustered mainly around lower probability scores. DAT, Dementia of the Alzheimer's type; pMCI, progressive mild cognitive impairment; pNC, progressive normal control; sMCI, stable mild cognitive impairment; uNC, unstable normal control

In Table 4, we provide the classification accuracy on the uNC, sMCI, pNC, pMCI, and eDAT independent test images computed using the 0.5 threshold. The classification accuracy on the eDAT images is the highest, followed by the uNC, sMCI, pMCI, and pNC in descending order. Figure 3 shows the distribution of the DAT probability scores for the uNC, sMCI, pNC, pMCI, and eDAT images as predicted by the final ensemble. The DAT− subgroups represented by the uNC and sMCI have low predicted DAT probability scores with mean values of 0.226 and 0.278, respectively. The DAT+ subgroups, however, show inconsistent distribution patterns. The eDAT subgroup has a single cluster of high‐predicted DAT probability scores with a mean value of 0.873, while the pMCI subgroup has two clusters of predicted DAT probability scores with an overall mean value of 0.623. Of the three DAT+ subgroups, the pNC subgroup has the lowest predicted DAT probability scores with a mean value of 0.261.

**Table 4:** Classification accuracy within each DAT− and DAT+ subgroup

Subgroups	Actual DAT−	Actual DAT+	Predicted DAT−	Predicted DAT+	Accuracy (%)
uNC	108	–	90	18	83.3
sMCI	871	–	653	218	75.0
pNC	–	56	44	12	21.4
pMCI	–	496	180	316	63.7
eDAT	–	238	25	213	89.5

Abbreviations: DAT, Dementia of the Alzheimer's type; pMCI, progressive mild cognitive impairment; pNC, progressive normal control; sMCI, stable mild cognitive impairment; uNC, unstable normal control.

**Figure 4:**
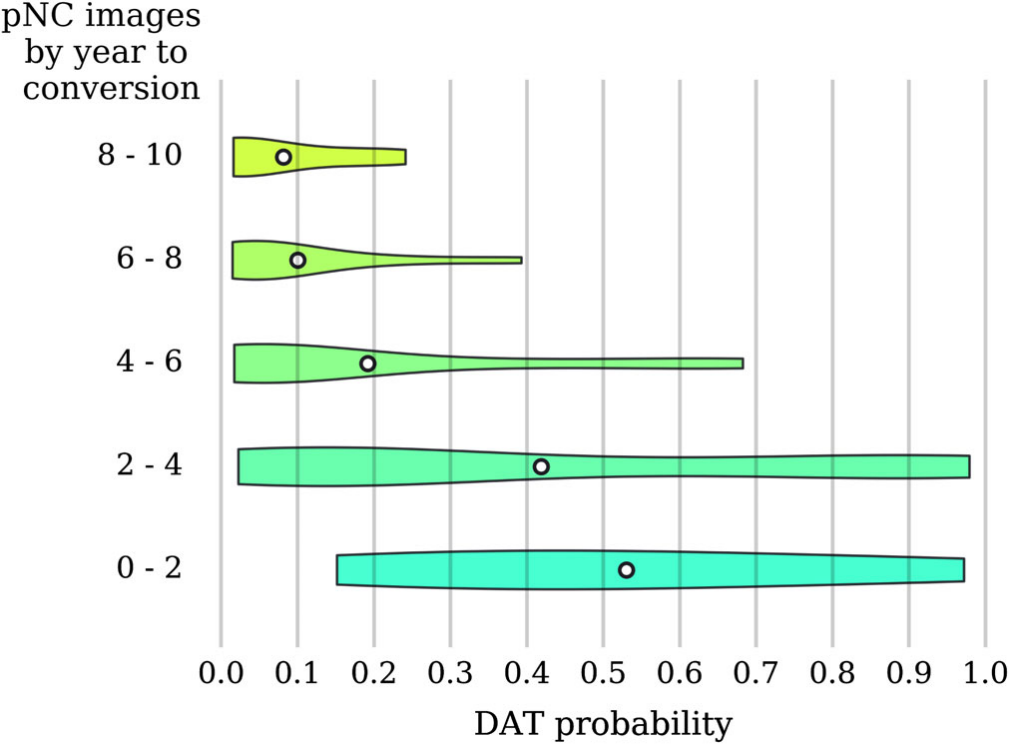
DAT probability scores across pNC images arranged by the time to conversion. The violin plot shows the density (relative proportion of images), while the white dot gives the mean probability. Note that for images taken closer to the time of conversion, there is a trend toward higher probability scores. DAT, Dementia of the Alzheimer's type; pNC, progressive normal control

**Figure 5:**
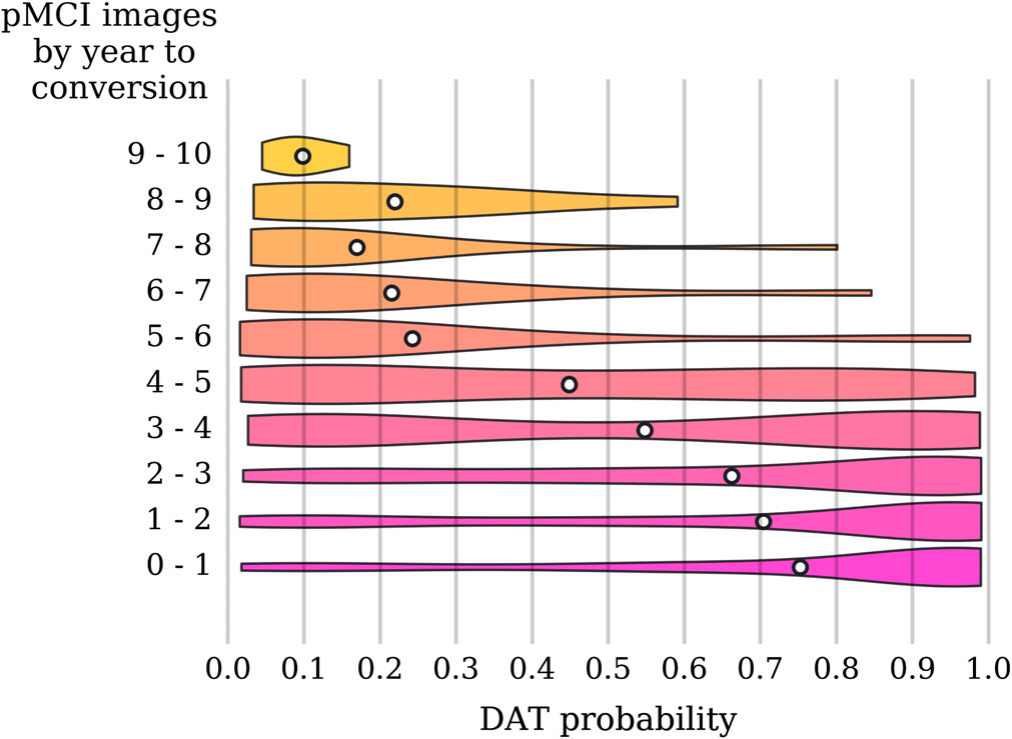
DAT probability scores across pMCI images sorted by the time to conversion. The violin plot shows the density (relative proportion of images), while the white dot gives the mean probability. Note that for images taken closer to the time of conversion, there is a trend toward higher probability scores. Prior to 5 years before conversion, there is more clustering around lower probability scores, and within 5 years before conversion, there is more clustering around higher probability scores, with a transition zone between years 3 and 5. DAT, Dementia of the Alzheimer's type; pMCI, progressive mild cognitive impairment

The pMCI and pNC are heterogeneous subgroups with varying disease severity and time to conversion. The number of years elapsed from an image scan date to a follow‐up date at which the pMCI or pNC subject's clinical diagnosis was changed to DAT is termed the time to conversion. In Figures 4 and 5, the predicted DAT probability scores when sorted by the time to conversion show a trend toward higher values as the time to conversion decreases. As expected, the DAT probability scores increase as the subjects approach conversion to a clinical diagnosis of DAT. Note that larger time to conversion ranges were used for the pNC images because the number of available pNC images is much smaller (see Table 1). The predicted DAT probability scores for the pMCI images with conversion time of 3 years are relatively high compared to those with conversion time exceeding 4 years. For the pNC images, however, even images with conversion time of 2 years have very low DAT probability scores. In Table 5, we list the classification accuracy as a function of years to conversion. We compared the performance of our method in the task of predicting MCI to DAT conversion in Table 6. Considering the large sample size in our experiment, the results show a clear advantage in accuracy and specificity for predicting MCI to DAT conversion, but offer a slightly lower sensitivity. Overall, our network achieved 0.793 AUC, 72.6% accuracy, 68.5% sensitivity, and 75.9% specificity on a completely independent and unseen test set consisting of the uNC, sMCI, pNC, pMCI, and eDAT images.

**Table 5:** Classification accuracy as a function of years to conversion

Subgroup	Year to conversion	Accuracy (%)
pNC	0–2	50.0
	2–4	38.9
	4–6	16.7
	6–8	0.0
	8–10	0.0
pMCI	0–1	80.2
	1–2	73.0
	2–3	68.5
	3–4	55.8
	4–5	45.5
	5–6	12.5
	6–7	10.0
	7–8	9.1
	8–9	11.1
	9–10	0.0

**Table 6:** Comparison of published sMCI versus pMCI classification performance evaluated on independent test set with 0–3 years time to conversion

Study	sMCI images	pMCI images	AUC	Accuracy (%)	Sensitivity (%)	Specificity (%)
Young et al. (2013)	96	47	0.767	69.9	**78.7**	65.6
Lange et al. (2016)	181	60	0.746	68.0	70.0	68.0
Popuri et al. (2018)	881	362	0.796	–	–	–
Our method	871	362	**0.811**	**74.7**	74.0	**75.0**

Abbreviations: pMCI, progressive mild cognitive impairment; sMCI, stable mild cognitive impairment.

### 4.3 Saliency and class activation maps

**Figure 6:**
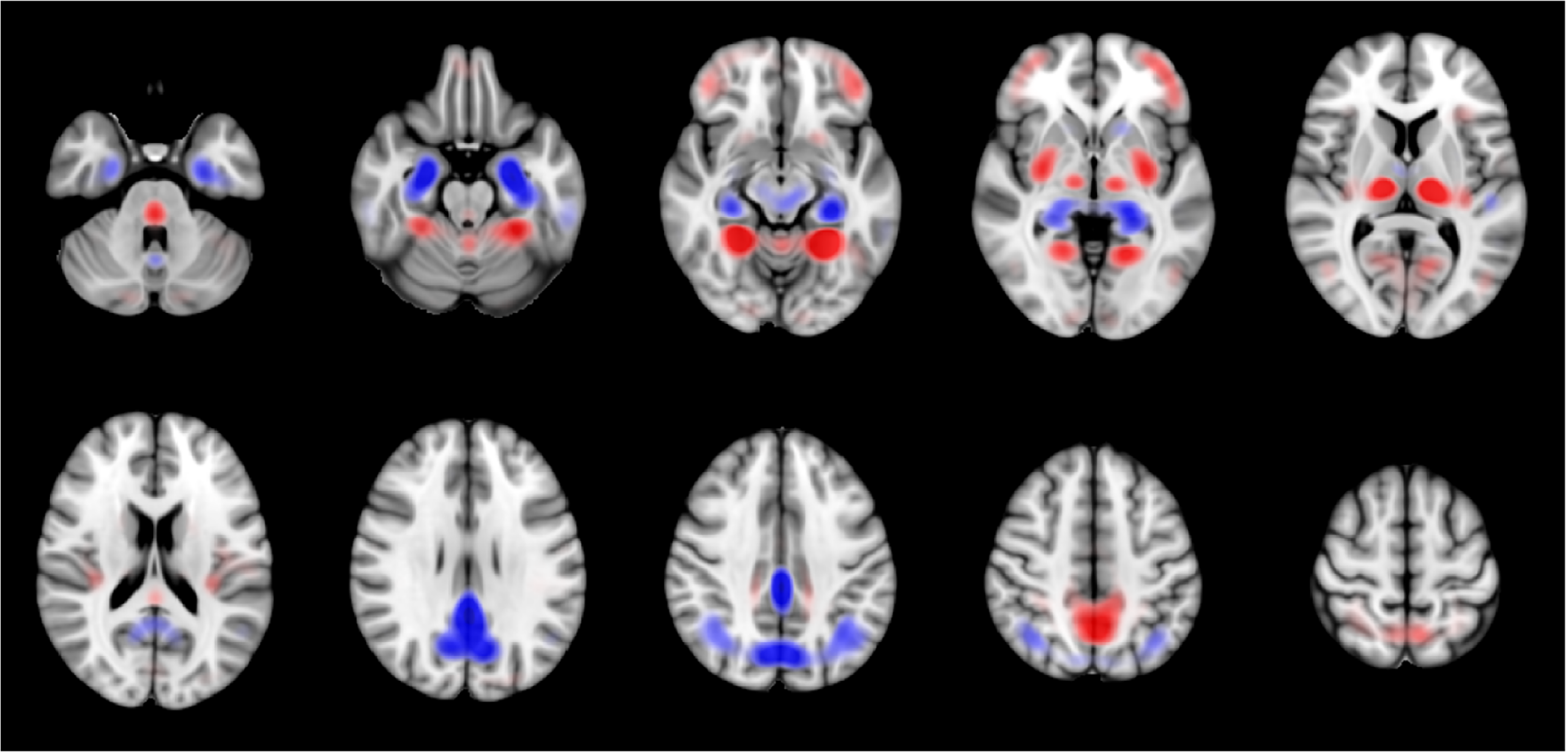
Grand average DAT saliency map. Regions with negative influence on the DAT class such that decreased intensities (blue) lead to higher predicted DAT probability score include the posterior cingulate cortex, middle cingulate cortex, angular gyrus, and hippocampus. Regions with positive influence on the DAT class such that increased intensities (red) lead to higher predicted DAT probability score include the thalamus, putamen, lingual gyrus, fusiform, ventral medial prefrontal cortex, pons and cerebellum. DAT, Dementia of the Alzheimer's type

**Figure 7:**
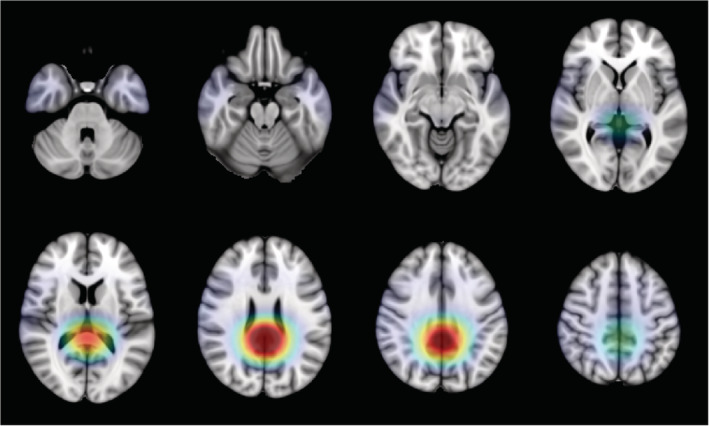
Grand average DAT class activation map highlighting the posterior cingulate cortex as the region most important for predicting the DAT class. DAT, Dementia of the Alzheimer's type

**Figure 8:**
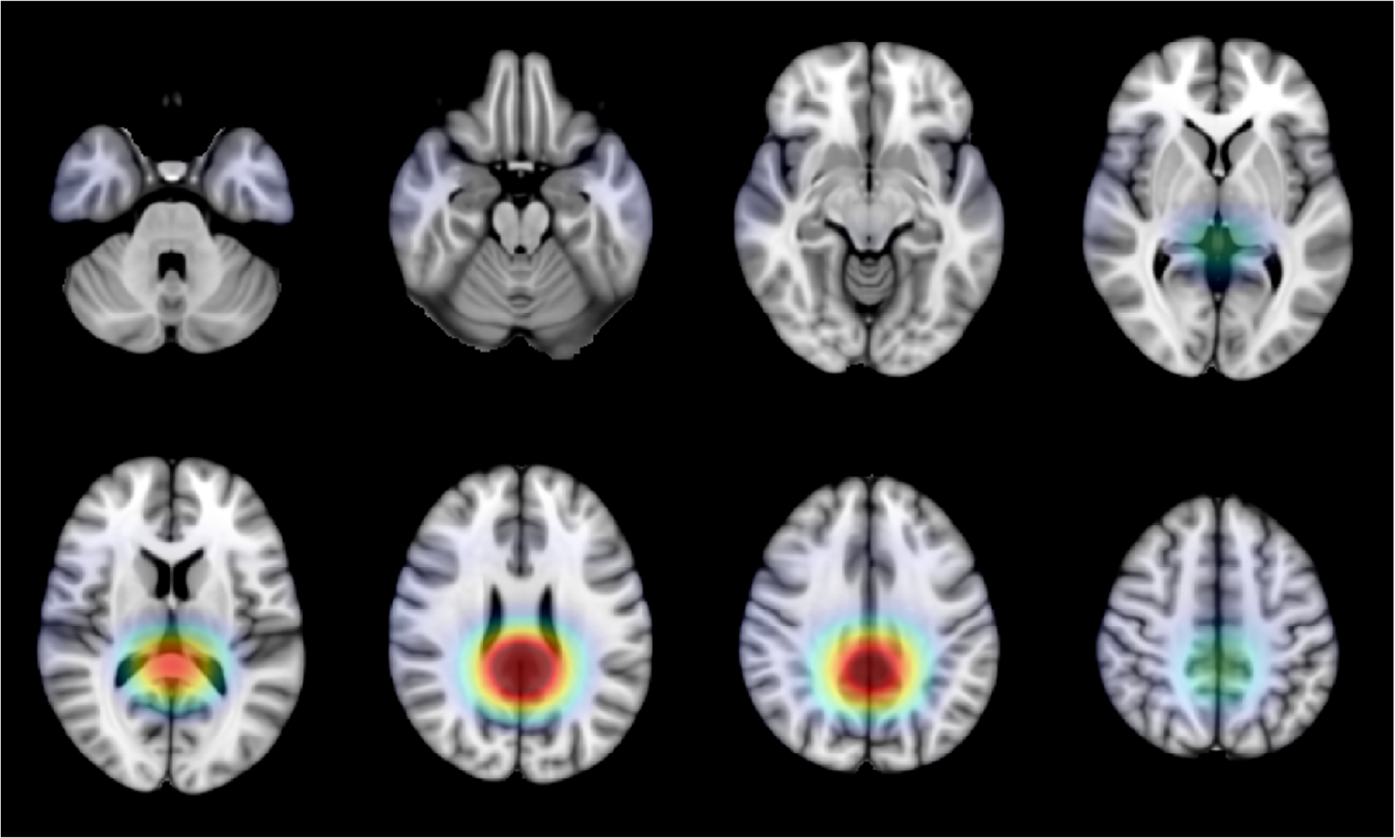
Grand average NC class activation map highlighting the cerebellum as the region most important for predicting the NC class. NC, normal control

The grand average DAT saliency map, which shows how small changes in the intensities of the input image affect the predicted DAT probability score, is presented in Figure 6. Regions with negative influence on the DAT class such that decreased intensities lead to higher predicted DAT probability score include the posterior cingulate cortex, middle cingulate cortex, angular gyrus, and hippocampus. Regions with positive influence on the DAT class such that increased intensities lead to higher predicted DAT probability score include the thalamus, putamen, lingual gyrus, fusiform, ventral medial prefrontal cortex, pons, and cerebellum. The precuneus shows negative influence on the DAT class except for its dorsal anterior subdivision which shows positive influence. The grand average DAT class activation map in Figure 7 highlights the importance of the posterior cingulate cortex for predicting DAT, while the grand average NC class activation map in Figure 8 highlights the importance of the cerebellum for predicting NC.

## 5 DISCUSSION

In this work, we presented a 3D CNN approach for classifying DAT using only FDG‐PET images. No associated MRI images are needed, and hence this method is more closely tuned to the clinical setting where one image (FDGPET) has been acquired and is being assessed and interpreted toward a clinical diagnosis of DAT. Throughout this work, we avoided common pitfalls that can lead to overly optimistic results. We performed repeated fivefold cross‐validation to ensure that our performance estimation is not sensitive to the choice of training samples. This cross‐validation strategy also allowed us to evaluate our model on a larger test set (20%). Importantly, we split the images at the subject level, ensuring that the training and test sets contained mutually exclusive subjects. Our model performed well on images from the extreme ends of the DAT spectrum, achieving 93.5% cross‐validated accuracy when tested on large number of sNC and sDAT images. Images observed in a real‐world clinical setting, however, can come from anywhere along the entire DAT spectrum. Of particular importance is the ability to accurately predict future conversion to DAT in those at the MCI stage, or even in those who are NC but are on the DAT+ trajectory. When tested on the uNC, sMCI, pNC, pMCI, and eDAT images, our model accuracy was 72.5%. As shown in Figure 3, the reduced performance is mainly due to misclassified pNC and pMCI images. In general, our model failed to predict conversion in pNC images across all conversion time ranges. Our model is, however, able to classify the pMCI images that are within 3 years of conversion to DAT with 74.0% accuracy. Further validation on an entirely independent clinical cohort is needed to verify that our model can handle scans obtained with different scanner parameters.

Visual interpretation is an important element of automated image analysis methods because it provides additional context around which the scoring of the image patterns is constructed. To better interpret the trained network prediction models, we analyzed the saliency and class activation maps. The saliency map in Figure 6 suggests that hypometabolism within the posterior cingulate cortex, precuneus, angular gyrus, and hippocampus is associated with DAT. This is consistent with the classic pattern of impaired metabolism observed in DAT (Del Sole et al., 2008; Jagust, Reed, Mungas, Ellis, & Decarli, 2007; Mosconi et al., 2005; Mosconi et al., 2008; Sanabria‐Diaz, Martínez‐Montes, & Melie‐Garcia, 2013). The patterns of hypermetabolism observed in Figure 6 echo those reported in a recent study (Katako et al., 2018). Although hypermetabolism is seldom reported in association with DAT, the regions showing hypermetabolism have been found to exhibit structural atrophy in DAT. Decreased volumes of the putamen and thalamus in DAT have shown significant correlation with cognitive test scores (De Jong et al., 2008). Volume loss in the fusiform is reported to occur at a higher rate of change in DAT and MCI (Holland et al., 2009). Moreover, thickness change in the fusiform is found to be predictive of cognitive decline on memory‐specific tasks (Murphy et al., 2010). A possible explanation for the observed hypermetabolism is that it may be a compensatory mechanism being recruited to preserve function in the face of network degradation due to AD. In their investigation of the relationship between glucose metabolism and memory function, Habeck et al. (2012) observed frontal hypermetabolism in DAT that is associated with better memory performance. Hypermetabolism might reflect an increased recruitment of neurons that serves to compensate for other affected areas. Even though averaging the saliency maps across all sNC and sDAT subjects should help reduce noise, it should be cautioned that the saliency map may still contain artifacts caused by discontinuities in the gradients.

Compared to the saliency map, the class activation maps in Figures 7 and 8 lack the spatial resolution required to discern voxel‐wise activity due to smoothing and upsampling. However, the class activation maps do highlight the region most important for predicting each target class. The DAT class activation map, as expected, highlights the posterior cingulate cortex, while the NC class activation map surprisingly features the cerebellum. In typical FDG‐PET analysis, metabolism is quantified only in relative terms and cerebellar metabolism is often used for intensity normalization. There have been inconsistent findings of DAT‐related metabolism in the cerebellum. An earlier study has found reduced metabolism in the cerebellum, while a more recent study has found increased metabolism in the cerebellum (Ishii et al., 1997; Mattis et al., 2016). Interestingly, cerebellar metabolism is reported to correlate with deficits in memory performance and social skills (Newberg et al., 2003). Overall, the spatial patterns captured by our 3D CNN are consistent with the literature.

A key motivation of our work was to develop a method for single modality imaging. However, the use of multimodality imaging and even nonimaging data may provide complementary information that can help improve predictive performance. Our method can be easily extended to train on multimodal images, which could help further improve prediction farther away from time to conversion.

## 6 CONCLUSIONS

We demonstrated the viability of using 3D CNN applied on FDG‐PET images for the classification of DAT and demonstrated the validity of the spatial patterns captured by our model. We also showed detailed performance metrics, focusing on a realistic performance evaluation that attempts to mimic a real‐world clinical situation. While our model achieved state‐of‐the‐art performance on classification of images along the entire DAT spectrum, it showed limited prognostic value in predicting future conversion to DAT using only an FDG‐PET image. Improvements can come from incorporating other imaging modalities and clinical nonimaging data into this flexible framework.

## Data Availability

The ADNI data used in this study is publicly available at http://adni.loni.usc.edu/.
